# Magnetic Separation and Centri-Chronoamperometric Detection of Foodborne Bacteria Using Antibiotic-Coated Metallic Nanoparticles

**DOI:** 10.3390/bios11070205

**Published:** 2021-06-23

**Authors:** Mohamed Fethi Diouani, Maher Sayhi, Zehaira Romeissa Djafar, Samir Ben Jomaa, Kamel Belgacem, Hayet Gharbi, Mihai Ghita, Laura-Madalina Popescu, Roxana Piticescu, Dhafer Laouini

**Affiliations:** 1 Laboratory of Epidemiology and Veterinary Microbiology (LEMV), Institut Pasteur de Tunis, LR11IPT03, Tunis-Belvédère 1002, Tunisia; sary14rev01@yahoo.com (S.B.J.); kamelbelgacem85@gmail.com (K.B.); ipthayetgharbi@Gmail.com (H.G.); 2 Campus Universitaire Farhat Hached B.P. n° 94-ROMMANA, Université Tunis El Manar, Tunis 1068, Tunisia; 3 Faculté des Sciences de Tunis, Campus Universitaire, El Manar, Tunis 2092, Tunisia; dhafer.laouini@pasteur.tn; 4 Laboratory of Transmission, Control and Immunobiology of Infections (LTCII), Institut Pasteur de Tunis, LR11IPT02, Tunis-Belvédère 1002, Tunisia; 5 Laboratory of Improvement and Development of Plant and Animal Production (ADPVA), Sétif 19000, Algeria; romeis.djafar@gmail.com; 6 Faculty of Sciences, University of Sétif, Sétif 19000, Algeria; 7 Faculté des Sciences de Bizerte, Université de Carthage, Bizerte 1054, Tunisia; 8 National R&D Institute for Non-Ferrous and Rare Metals, INCDMNR-IMNR, 102 Biruintei Blvd, Pantelimon, 077145 Ilfov, Romania; mihai@imnr.ro (M.G.); mpopescu@imnr.ro (L.-M.P.); roxana.piticescu@imnr.ro (R.P.)

**Keywords:** centri-chronoamperometry, biosensor, nanoparticles, foodborne bacteria, antibiotic

## Abstract

Quality and food safety represent a major stake and growing societal challenge in the world. Bacterial contamination of food and water resources is an element that pushes scientists to develop new means for the rapid and efficient detection and identification of these pathogens. Conventional detection tools are often bulky, laborious, expensive to buy, and, above all, require an analysis time of a few hours to several days. The interest in developing new, simple, rapid, and nonlaborious bacteriological diagnostic methods is therefore increasingly important for scientists, industry, and regulatory bodies. In this study, antibiotic-functionalized metallic nanoparticles were used to isolate and identify the foodborne bacterial strains *Bacillus cereus* and *Shigella flexneri*. With this aim, a new diagnostic tool for the rapid detection of foodborne pathogenic bacteria, gold nanoparticle-based centri-chronoamperometry, has been developed. Vancomycin was first stabilized at the surface of gold nanoparticles and then incubated with the bacteria *B. cereus* or *S. flexneri* to form the AuNP@vancomycin/bacteria complex. This complex was separated by centrifugation, then treated with hydrochloric acid and placed at the surface of a carbon microelectrode. The gold nanoparticles of the formed complex catalyzed the hydrogen reduction reaction, and the generated current was used as an analytical signal. Our results show the possibility of the simple and rapid detection of the *S. flexneri* and *B. cereus* strains at very low numbers of 3 cells/mL and 12 cells/mL, respectively. On the other hand, vancomycin-capped magnetic beads were easily synthesized and then used to separate the bacteria from the culture medium. The results show that vancomycin at the surface of these metallic nanoparticles is able to interact with the bacteria membrane and then used to separate the bacteria and to purify an inoculated medium.

## 1. Introduction

Foodborne infectious diseases represent some of the most common public health problems that generate an enormous social and economic burden worldwide [1,2]. These diseases are mostly linked to the existence of microorganisms and toxins in food and water [3]. Over 250 different foodborne illnesses that can lead to severe diarrhea, debilitating infections, severe poisoning, long-term illness, lasting disability, or even death have been identified [4]. Thus, pathogens causing such diseases represent a real source of human suffering.

In 2017, bacterial agents were found to be responsible for the most reported foodborne (including waterborne) outbreaks in the European Union, with 50.4% of the total outbreaks. Specifically, 34.3% of the outbreaks were due to bacteria, 16.1% were caused by bacterial toxins, while 7.8%, 3.6%, and 0.6% implicated viruses, other causative agents, and parasites, respectively [5].

In addition, it has been reported that 1.6 million people died from diarrheal diseases in 2016 globally, and around half a million of them are children aged 5 years and younger. A large proportion of these cases is attributed to the contamination of food and drinking water [6].

*B. cereus* and *Shigella* are tremendous etiological agents of human diarrheal syndrome worldwide [7,8]. *B. cereus* are spore-forming Gram-positive bacteria that are capable of forming endospores and producing enterotoxins, responsible for the diarrheal syndrome, and emetic toxins, the causative agents of the emetic syndrome [9]. However, *Shigella* are non-spore-forming Gram-negative bacteria that cause a disease called Shigellosis or bacillary dysentery, which is responsible for 165 million diarrheal episodes and 600,000 deaths each year worldwide [10,11]. The latter are highly transmissible pathogens, and some of their strains can cause active infections in humans with exposure to only 10 cells [8,12]. Hence, there is a need to find ways to detect these bacteria at very low doses.

Certain conventional and modern diagnostic tools are described as inexpensive and provide both qualitative and quantitative information on the tested microorganisms. However, these tools are above all limited by the duration or the time suitable for the identification of pathogens, as well as by the sensitivity and the high detection limit [13,14,15].

Advances in technology and technical revolutions have enabled the development of new tools that largely meet current medical requirements. Biosensors are devices that detect a biological or physiological event and convert it into a measurable, quantifiable, and easy-to-use physical signal [16,17]. In the health field, these biosensors have multiple applications, in particular for the rapid detection and identification of pathogens at early stages of infection. To this end, many biosensors have been reported for the rapid and sensitive detection of foodborne bacteria and other pathogens [18,19].

Recently, nanotechnology has played a very important role in the improvement of these miniature biosensing systems. The introduction of nanomaterials into biosensors has remarkably improved the sensitivity, specificity, and selectivity of these devices [20,21]. In addition, these nanomaterials have reduced the required time to detect pathogens and have eliminated cumbersome steps in the diagnostic process [22,23,24]. Metallic nanoparticles have been proposed for these biomedical purposes [25]. During recent years, both gold and magnetic nanoparticles have been coupled with various biological molecules, including different types of antibiotics [26,27,28,29,30]. Equally, with their good biocompatibility, the unique physical, chemical, optical, and electrical properties of these materials have increased their range of applications [25,31,32,33,34]. The superparamagnetic propriety of iron oxide nanoparticles makes them a good candidate in many applications such as drug targeting, pathogen and toxin separation, and media purification [35,36,37]. Likewise, gold nanoparticles were widely used in cell targeting and labeling and pathogen detection based on their optical properties and electrochemical catalytic activity [38,39,40].

In the present study, antibiotic-coated iron oxide magnetic nanoparticles were employed in order to easily extract and separate foodborne bacteria, to concentrate them in small volumes, and, by consequence, to assess the purification process of bacteria-inoculated liquid media. Separately, vancomycin-coated gold nanoparticles were employed to develop a centri-chronoamperometric assay for the rapid and easy detection of two foodborne bacterial strains, *B. cereus* and *S. flexneri*. This system is of great interest in the bacterial sensing field, taking advantage of the ability of vancomycin at the surface of the nanoparticles to interact with a broad range of Gram-positive and Gram-negative bacteria. The use of gold nanoparticles allowed the easy and rapid labeling of bacteria in suspension with electrochemical detectable materials. Bacteria detection was performed without the need of a preimmobilization step of bioreceptors on the surface of the transducer, taking advantage of the gold labeling and the electrocatalytic activity of these metallic nanoparticles toward the reduction of hydrogen ions at the surface of a polarized immobilization-free carbon electrode.

## 2. Experimental Section

### 2.1. Reagent and Materials

LB agar growth medium was obtained from Merck, France, and was used to replicate *S. flexneri* (*ATCC 29903*) and *B. cereus* (*ATCC 11768)*, available as reference strains from the Laboratory of Epidemiology and Veterinary Microbiology at Pasteur Institute of Tunis. LB broth bacterial culture medium was also obtained from Merck. Two antibiotics, vancomycin and penicillin, were purchased from Medis, Tunis, Tunisia, and Biochemie GmbH, Vienna, Austria, respectively. Chloroauric acid (HAuCl_4_·4H_2_O), ammonium iron (II) sulfate hexahydrate (NH_4_)_2_Fe (SO_4_)_2_·6H_2_O, ammonium iron (III) sulfate dodecahydrate (NH_4_Fe (SO_4_)_2_·12H_2_O), ammonium hydroxide (NH_4_OH), and sodium hydroxide (NaOH) were purchased from Sigma-Aldrich, St. Quentin Fallavier, France.

Phosphate-buffered saline (PBS 0.1 M, pH 7.4) was prepared by mixing 8 g of sodium chloride (NaCl), 0.2 g of potassium dihydrogen phosphate (KH_2_PO_4_), 1.53 g of disodium hydrogen phosphate dodecahydrate (Na_2_HPO_4_·12H_2_O), and 0.2 g of potassium chloride (KCl) in 1 L of distilled water. The pH value was adjusted with a Thermo Electron Corporation (Waltham, MA, USA) pH meter using hydrogen chloride (HCl) or sodium hydroxide (NaOH).

A UV–vis spectrophotometer (Single-Beam LI-295, Lasany, India) was used during this study to characterize the synthesized AuNP@vancomycin and to measure the optical density of the bacteria-inoculated media.

Scanning electron microscopy images were taken with a scanning electron microscope (GSED-SEM) operated at 15.0 kV. Electrochemical measurements were carried out by using the potentiostat Voltalab 40 PGZ 301 model from Radiometer Analytical Instrument S.A. (Loveland, CO, USA), which is controlled by VoltaMaster 4 software. A three-electrode electrochemical cell (screen-printed carbon electrodes (SPCEs) DRP-110) was obtained from DropSens (Llanera, Asturies, Spain).

### 2.2. Bacterial Culture

Two bacteria strains, *B. cereus* (*ATCC 11768*) and *S. flexneri* (*ATCC 29903*), were prepared by culturing in both solid (agar) and liquid (LB broth) media in order to confirm the viability of the bacteria and to obtain a pure and good volume of the bacterial suspensions. The bacterial growth in LB media was evaluated by the presence of turbidity during the visual reading of the tubes, and the bacterial cell count was statistically estimated using the most-probable-number (MPN) method for a three-tube assay with a 10-fold dilution [41,42]. Consequently, and by referring to the MPN table with the 95% confidence interval [43,44], the *B. cereus* concentration was estimated to be 93 cells/mL (95% CI: 18–360 cells/mL), while the *S. flexneri* concentration was 23 cells/mL (95% CI: 4.6–94 cells/mL).

### 2.3. MNP@antibiotics Synthesis

Antibiotic-functionalized iron oxide magnetic nanoparticles were synthesized using the chemical coprecipitation method of iron(II) and iron(III) in alkaline medium [45,46]. Namely, 10 mg of antibiotics (penicillin or vancomycin) was dissolved in 25 mL of distilled water and 1 mL of ammonium hydroxide (NH_4_OH, 25%). Then, 25 mL of previously prepared iron solution (0.5 g of ammonium iron(II) sulfate hexahydrate and 0.615 g of ammonium iron(III) sulfate dodecahydrate (iron(II) and (III): molar ratio = 1:1) in 25 mL of distilled water) was added drop by drop at room temperature under vigorous stirring. The pH of the solution was adjusted to 12 using sodium hydroxide (NaOH, 2 M). After 30 min, a black precipitate was obtained, indicating the formation of superparamagnetic particles, and separated by a magnet. The nanoparticles were repeatedly washed (5 times) with deionized water to eliminate the unbounded antibiotics and obtain a suspension of MNP@antibiotics at neutral pH. Finally, a drop of the synthesized MNP@antibiotic solution was placed on a silica wafer and then left to dry at room temperature. The wafer was placed in the scanning electron microscope to make the necessary analyses.

### 2.4. MNP@antibiotics Agglutination Assay

Into each well of a clean glass slide, a drop of 40 µL of bacterial suspension (*B. cereus* or *S. flexneri*) was mixed with an equal volume of a previously synthesized MNP@antibiotic (MNP@vancomycin or MNP@penicillin) dispersed in PBS. The mixtures were spread a little with clean pipette tips. Gentle rocking movements were carried out for 30 min at room temperature. The results were visually examined with the naked eye and a binocular magnifier. For the negative control, MNP@antibiotic was mixed with noninoculated liquid medium. The interaction between the antibiotic and the bacteria resulted in the formation of an NPM/bacteria complex. The nanoparticles surround the bacteria by the attachment of the antibiotic to its target molecules at the cells’ surfaces. Some nanoparticles attracted more than one pathogen cell to form a network of bacteria surrounded by nanoparticles, which was observed on the slide as connected black buttons. In the absence of an affinity between the antibiotic and the bacteria, no MNP/bacteria complex was formed. The nanoparticles remained free in the solution, and consequently, no changes were observed.

### 2.5. Antibacterial Activity of MNP@vancomycin against B. Cereus

Two-fold serial dilutions with a starting concentration of 0.5 mg/mL of vancomycin-coated magnetic nanoparticles in PBS were performed in sterile tubes. A volume of 1 mL was obtained in each tube. Then, 9 mL of *B. cereus* suspension was added to each tube. Next, the mixtures were placed in an oven at 37 °C for 2 h with gentle stirring. The bacteria were attached to the surface of the nanoparticles to form MNP@vancomycin/*B. cereus* complexes. These complexes were captured and isolated through a magnet by exposing the tubes to an external magnetic field. Finally, and after the separation of the nanoparticles, 1 mL of the remaining solution was placed in a quartz cuvette and then placed in the spectrophotometer to measure the optical density at a wavelength of 600 nm.

### 2.6. AuNP@vancomycin Synthesis and Characterization

Vancomycin-functionalized gold nanoparticles were synthesized as described by [47]. Mainly, in a flask covered with aluminum foil to protect its contents against light, 0.6 mM HAuCl_4_.3H_2_O, 0.3 mM vancomycin, and 7 mM NaOH were mixed in a total volume of 50 mL of distilled water. The pH of the obtained solution was measured as 10.5. Then, the solution was incubated at 80 °C with stirring for 20 h until the color changed to dark red, which indicated the formation of gold nanoparticles. Furthermore, the solution was allowed to cool at room temperature and then centrifuged at 4500× *g* for 10 min to remove the excess of the antibiotic. The supernatant was recovered and centrifuged again at 14,000× *g* for 10 min. Finally, the pellet was recovered, suspended in PBS, and then stored at +4 °C until use. The characterization of these nanoparticles was carried out by measuring its absorption spectrum between 300 nm and 700 nm using a UV–visible spectrophotometer. The presence of nanoparticles was characterized by the appearance of an absorption band at a wavelength around 520 nm.

Moreover, electrochemical characterization of the synthesized AuNP@vancomycin was carried out by testing the ability of gold nanoparticles to electrocatalyze the hydrogen evolution reaction. For this purpose, 1 mL of AuNP@vancomycin was centrifuged in an Eppendorf tube at 14,000× *g* for 10 min. The supernatant was removed, and the recuperated nanoparticles in the pellet were dispersed in 25 µL of PBS and 25 µL of 2 M HCl. Finally, the mixture was transferred at the surface of a screen-printed carbon electrode polarized at +1350 mV for 60 s followed by −1000 mV for 100 s, and the generated cathodic current was recorded during this step. For the negative control, the same step was repeated with 25 µL of PBS and 25 µL of 2 M HCl without the use of gold nanoparticles.

### 2.7. Electrochemical Detection of Bacteria

In Eppendorf tubes, 1 mL of different bacterial concentrations were prepared from 2-fold cascade dilutions with an initial suspension of *B. cereus* at 93 cells/mL. Then, the obtained bacterial suspensions were centrifuged at 4000× *g* for 10 min. The supernatant was removed, the pellet was recovered, and 1 mL of AuNP@vancomycin was added to each tube. The mixture was gently agitated for 30 min to form an AuNP@vancomycin/bacteria complex by the interaction of the vancomycin at the gold nanoparticle surface with its binding site at the bacterial cell wall. Next, centrifugation at 4000× *g* for 10 min was carried out to bring this complex down. In fact, only the nanoparticles attached to the bacteria were recovered in the pellet. Nonattached nanoparticles remained dispersed at this centrifugation speed, and they were eliminated with the supernatant. Finally, to the collected pellets, 25 μL of PBS and 25 μL of 2 M HCl were added, and a sonication step was carried out for 10 min. Then, this nanoparticles solution in acidic medium was deposited at the surface of a miniature flat electrochemical cell connected to a VoltaLab 40 potentiostat, PGZ301. Chronoamperometric curves were recorded by measuring the generated current after applying a potential of +1350 mV for 60 s, followed by 100 s at –1000 mV.

To test the ability of this biosensor to detect other vancomycin-sensible bacteria, the same procedure was repeated using 2-fold cascade dilutions of the suspension of *S. flexneri* with an initial concentration of 23 cells/mL. For the negative control, noninoculated LB broth culture medium was used.

## 3. Results and Discussion

### 3.1. Characterization of the Antibiotic Stabilized Magnetic Nanoparticles

**Figure 1:**
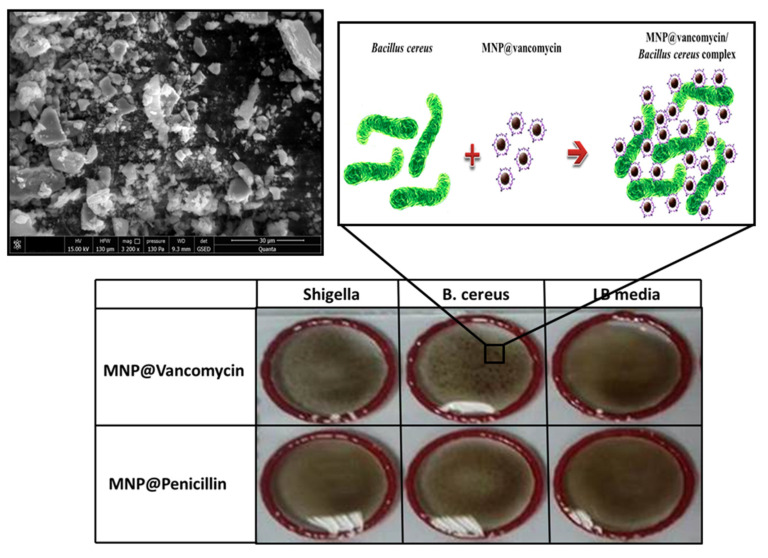
Scanning electron microscope image of the synthesized vancomycin-coated iron oxide magnetic nanoparticles (**upper left** panel). Schematic illustration of the MNP@antibiotic agglutination test (**upper right** panel). Evaluation of the interaction between two antibiotics, penicillin and vancomycin, stabilized magnetic beads with the two bacterial strains, *B. cereus* and *S. flexneri*, using the MNP@antibiotic agglutination test (**lower** panel).

Unlike other metallic nanoparticles (copper, silver, or zinc), iron oxide nanoparticles do not have high antimicrobial effects. One study, performed on pure iron oxide nanoparticles, suggests that these particles are known for their limited antibacterial activity [48]. Hence, many studies are interested in finding a way to functionalize them with molecules or antibacterial agents. In this work, vancomycin-coated iron oxide magnetic nanoparticles were synthesized using the chemical coprecipitation method. Scanning electron microscope images of the obtained Fe_3_O_4_@vancomicin sample are shown in Figure 1. The results show varied particle sizes ranging from a few nanometers to aggregates of around 20 µm. Moreover, the SEM images show variability in the shape of these particles.

In addition, an MNP@antibiotic agglutination test was performed in order to evaluate both the presence of the antibiotic at the surface of the nanoparticles and the ability of these coated nanoparticles to interact with the cell wall surfaces of bacteria. The mixing of an antibiotic-attached MNP with a bacterial suspension induces an interaction between the antibiotic active site and the biomolecules, to which it has affinity, in the pathogen wall surface, leading to the formation of an MNP@antibiotic/bacteria complex (Figure 1). Because the bacterial size is larger than the size of the nanoparticles, many MNP@antibiotic complexes can bind to the bacteria membrane to form aggregates of particles at the bacterial surfaces. Furthermore, and due to their large surface area, some of the functionalized nanoparticles attract more than one bacterium, which is reflected in the formation of networks of bacteria surrounded by nanoparticles, hence the agglutination of bacteria under the action of MNP@antibiotic. This phenomenon is observed on the glass slide well as connected dark black granules.

In the present study, two bacteria, *B. cereus* and *S. flexneri*, were first mixed with vancomycin-coated MNPs. Figure 1 shows a strong agglutination translated by the appearance of granules in the first two slide wells of the first line corresponding to MNP@vancomycin. This positive agglutination allows inferring that vancomycin at the surface of the nanoparticles is able to interact with the biomolecules (its binding sites) presented on the cell wall of the two bacterial strains, *B. cereus* and *S. flexneri*.

In contrast, a negative agglutination was observed when bacteria were incubated with MNP@penicillin. Figure 1 shows that the nanoparticles remain dispersed, and no interaction took place between the antibiotic and the cell surfaces. This result may be explained by the fact that the two bacteria are penicillin resistant. It is known that *B. cereus* produces a large number of beta-lactamases; therefore, this molecule deactivates the antibiotic’s antibacterial properties through the breaking of its β-lactam ring by hydrolysis. The production of the beta-lactamase enzyme provides to *B. cereus* a resistance to penicillin as a β-lactam antibiotic. Similarly, previous studies have shown that several strains of *Shigella* have developed a resistance to penicillin [49,50]. This penicillin resistance was observed even in some *Shigella* strains that emerged before the antibiotic description in 1929 such as the historical *S. flexneri* isolate NCTC1, which was isolated in 1915 and found resistant to penicillin [51].

For the negative control, both antibiotic-coated nanoparticles were mixed with noninoculated LB broth culture medium. The results show that the nanoparticles remained dispersed, and no agglutination occurred in the slide wells. In conclusion, the agglutination test results show that the two tested bacteria, *B. cereus* and *S. flexneri*, attract MNP@vancomycin to bind to their cell walls.

### 3.2. Antibacterial Activity of MNP@vancomycin against B. Cereus

The interaction between MNP@vancomycin and the *B. cereus* strain was further investigated. In addition to their valuable and unique magnetic properties, the synthesized iron oxide nanoparticles are considered to be extraordinarily biocompatible, which makes them an ideal candidate for magnetic separation and sample purification. Our results show that the synthesized magnetite exhibits huge magnetization when applied to an external magnetic field. However, this magnetization disappears as soon as the magnetic field is stopped, and by consequence, no more magnetic interaction remains. This phenomenon, called superparamagnetism, allows the remote manipulation of the particles with external fields, which makes them useful for various medical applications such as cell separation and sample concentration.

**Figure 2:**
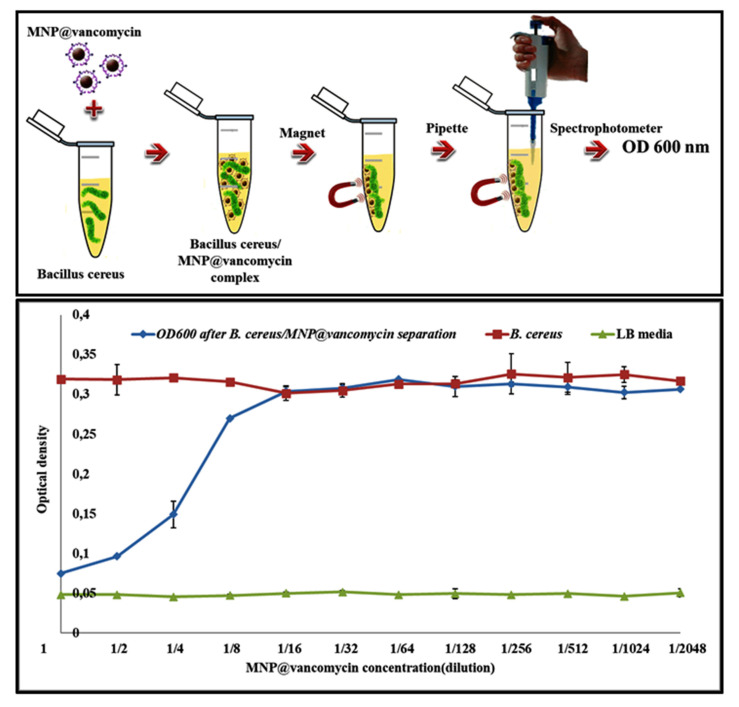
Schematic representation of the steps involved in bacterial separation and media purification using MNP@vancomycin (**upper** panel). Optical density response measured at 600 nm for a *B. cereus*-inoculated media (positive control) (red), noninoculated media (negative control) (green), and *B. cereus*-inoculated media after the magnetic separation of the bacteria using different concentrations of MNP@vancomycin (blue) (**lower** panel).

These particles were used to develop a quick and simple diagnostic method: MNP@antibiotic agglutination assay. This method could be applied to capture and detect a wide range of bacteria. Likewise, MNP@vancomycin was used in this work to separate the *B. cereus* strain and to easily purify the liquid LB media. As shown in Figure 2, these biofunctionalized nanoparticles have the ability to fix pathogens and then easily eliminate them from the media by applying an external magnetic field. Thus, media purification is strongly dependent on the nanoparticles’ concentration.

The interaction between the bacteria and the particles results in the formation of MNP@vancomycin/*B. cereus* complexes. As a consequence, the elimination of MNP@vancomycin from the media through a magnet is accompanied by the removal of the bacteria, which is translated by a decrease in the optic density at 600 nm. Figure 2 shows that the bacterial concentration is proportional to the particle concentration. After the incubation of the inoculated media with 0.5 mg/mL or 0.25 mg/mL of MNP@vancomycin, the obtained OD600 is almost similar to that obtained with noninoculated media, below 0.1. This value increases with the number of dilutions until it reaches the OD600 of *B. cereus*-inoculated media at a 1/16 nanoparticle dilution. Beyond this dilution, the OD600 was kept constant slightly higher than 0.3 and equal to the OD600 of the inoculated media, which indicates that the bacterial concentration was kept constant in the media, and the maximum bacterial growth was always reached. Therefore, MNP@vancomycin lose their antibacterial activity and then lose their ability to purify the liquid media.

### 3.3. Characterization of the Vancomycin-Functionalized Gold Nanoparticles

**Figure 3:**
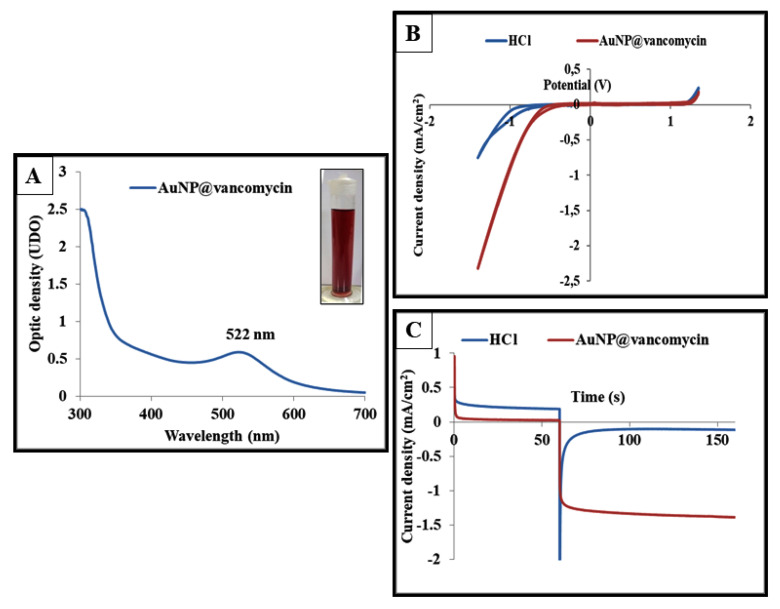
UV–visible absorption spectra and photographs of the vancomycin-coated gold nanoparticles (**A**). Cyclic voltammetry curves recorded between +1.35 V and −1.40 V at a scan rate of 50 mV/s for a 1 M HCl solution in the absence and in the presence of AuNP@vancomycin (**B**). Chronoamperograms were recorded by applying a potential of +1.35 V for 60 s, followed by −1.00 V for 100 s, in 1 M HCl solution without (**upper** curve) and in the presence (**lower** curve) of AuNP@vancomycin at the surface of a screen-printed carbon electrode (**C**).

In 2016, Hur and Park developed a one-step and one-pot green synthetic route process in order to synthesize spherically shaped AuNP@vancomycin with an average diameter of 11.01 ± 3.62 nm [47]. Here, this method was employed using aqueous chloroaurate ions as gold precursors and vancomycin as both reducing and capping agents. After 20 h of incubation at 80 °C, the solution color changed from yellow to dark red, indicating the reduction of the gold ions and the growth of gold nanoparticles (Figure 3A). This color was reached because of the phenomenon of the surface plasmon resonance, which induces the reflection of the red light when the gold particles are smaller than 30 nm. Then, the obtained product was characterized with a UV–visible spectrophotometer. As expected, Figure 3A shows a peak of absorbance appearing at 522 nm, which indicates the successful synthesis of the vancomycin-stabilized gold nanoparticles.

Moreover, these particles were further characterized electrochemically with cyclic voltammetry (Figure 3B) and by measuring the chronoamperometric signal generated due to the electrocatalytic activity of AuNPs toward the reaction of the reduction of hydrogen ions at the surface of a polarized electrode (Figure 3C).

The recorded voltammograms show an increase in the peak of proton reduction between −0.6 V and −1.4 V in the presence of AuNP@vancomycin in HCl solution. At the surface of an electrode, the gold nanoparticles catalyze the reduction of protons in acidic medium by applying an appropriate potential (for example, −1.00 V in the case of 1 M HCl) [52,53]. Furthermore, it was reported that this catalytic activity could be improved by a previous oxidation step of the gold nanoparticles at +1.35 V for 60 s. By this oxidative potential, some of the gold atoms from the surface of the nanoparticles were released in the media as Au (III) ions. The latter exert an additional catalytic activity as well as the rest of the nanoparticles [40,54,55,56].

In the present study, AuNP@vancomycin were deposited at the surface of a screen-printed carbon electrode, then a gold oxidative potential of +1.35 V was applied for 60 s, followed by proton reduction potentials of –1.00 V for 100 s. The chronoamperometric signals were recorded and are presented in Figure 3B. The results show an increase in the absolute value of the generated cathodic current in the presence of the AuNP@vancomycin. After 60 s of a gold preoxidation step and 100 s of proton reduction, the measured current was found to be −1.38668 mA/cm^2^ in the presence of AuNP@vancomycin. In contrast, this value was found to be very close to zero in the absence of these nanoparticles to be equal to −0.115446 mA/cm^2^. Thereby, the catalytic effect of the synthesized AuNP@vancomycin toward the hydrogen evolution reaction was successfully confirmed.

### 3.4. AuNP@vancomycin-Based Centri-Chronoamperometric Biosensor for Bacteria Detection

**Figure 4:**
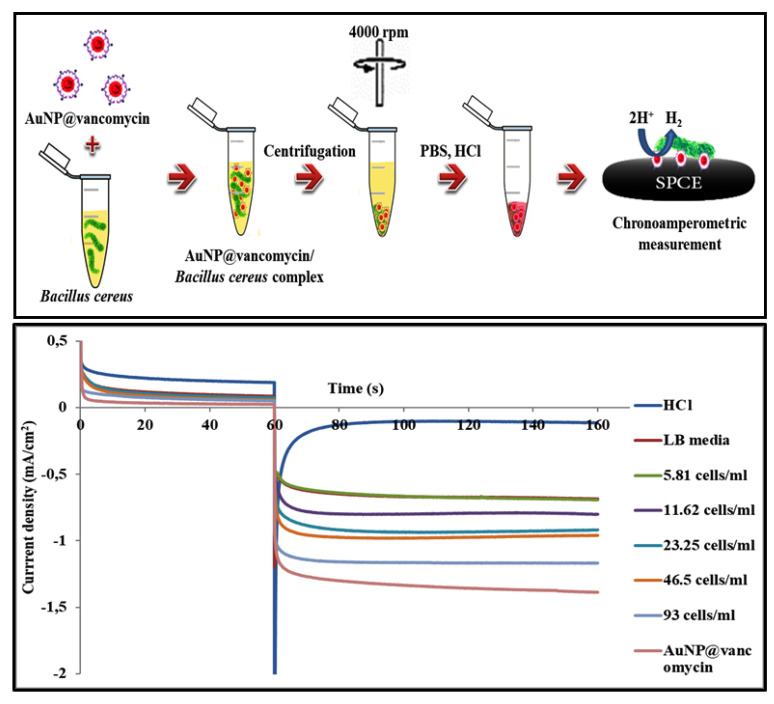
Schematic illustration of the strategy used to develop the vancomycin-coated gold nanoparticle-based centri-chronoamperometric assay for the rapid and sensitive detection of foodborne bacteria (**upper** panel). Chronoamperometric curves obtained without bacteria (LB media) and with 5.81; 11.62; 23.25; 46.5 and 93 cells/mL of the bacterial strain, *B. cereus* (**lower** panel).

Gold at the nanometric scale represents one of the most attractive metals due to its nobleness and its very interesting properties [33]. Nowadays, gold nanoparticles are widely employed in the targeting, labeling, and capture of biological molecules, cells, and organisms [38]. Here, a vancomycin-capped gold nanoparticle was used to label and detect the *B. cereus* strain by centri-chronoamperometric assay (Figure 4). As reflected, this method is made up of two key steps: centrifugation and chronoamperometry.

In the first step, the pathogens were labeled with an electrochemical active material, AuNP, and then extracted with the centrifugation method. To this end, the amount of nanogold present in the separated labeled bacteria is proportional to the number of extracted bacteria. Therefore, the detection of the presence of gold nanoparticles is large enough to note the existence of the pathogenic cells. This extraction method is very promising, taking advantage of the fact that the capture and the isolation of the bacteria were conducted in suspension, which allows the easy and quick interaction of AuNP@vancomycin with the biomolecules overexpressed on the surface of the bacteria.

In more detail, the heptapeptide backbone of vancomycin at the surface of the gold nanoparticles interacts with the D-alanyl-D-alanine dipeptide expressed at the bacteria wall surface through a five-hydrogen-bond motif [29,30,57]. As a result, an AuNP@vancomycin/*B. cereus* complex was formed. This complex was easily separated from the media by centrifugation. It is well known that the bacteria cells sediment in the bottom of a tube when centrifugation at 4000× *g* for 10 min is carried out. However, this rotation speed is insufficient for the gold nanoparticles’ separation due to their small sizes. Consequently, only gold nanoparticles that formed parts of the AuNP@vancomycin/*B. cereus* complex were recuperated after this extraction process. Other unbounded nanoparticles remained dispersed in the suspension and were eliminated by throwing out the supernatant.

In the second step, the detection of the pathogen was performed by detecting the amount of AuNPs fixed at the surface of the separated bacteria. This process was conducted by chronoamperometry thanks to the catalytic activity of the AuNPs. As described above, gold nanoparticles catalyze the reaction of proton reduction in acidic media. As a result, an increase in the generated cathodic current was obtained after the incubation of the AuNP@vancomycin/*B. cereus* complex with the polarized electrode. This generated AuNP-proportional current was used as the analytic signal in the present assay.

To evaluate the AuNP@vancomycin-based centri-chronoamperometric assay, suspensions with different *B. cereus* concentrations ranging from 93 cells/mL to cells/mL were prepared. Vancomycin-functionalized AuNPs were employed for the labeling of the bacterial cells, taking benefit from the interaction between the antibiotic and its binding site at the bacterial surface. The extraction of the bacteria was conducted with centrifugation, and the extracted AuNP-labeled bacteria were then detected by chronoamperometry as described.

The chronoamperometric-obtained signals are displayed in Figure 4. As observed, the absolute value of the generated current density is proportional to the bacterial concentration. For a *B. cereus* concentration of 93 cells/mL, the generated cathodic current was 1.13 ± 0.06 mA/cm^2^. This value was gradually decreased with a decrease in the bacterial concentration to reach only 0.73 ± 0.05 mA/cm^2^ with 6 cells/mL.

**Figure 5:**
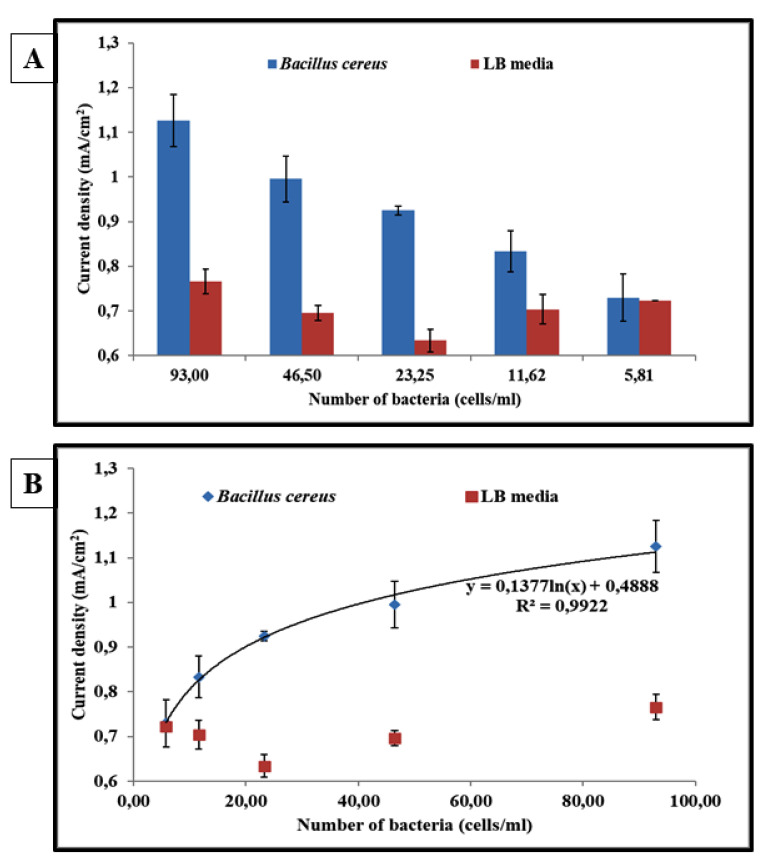
Diagrams correspond to the response of the centri-chronoamperometric assay to various *B. cereus* concentrations ranging from 6 cells/mL to 93 cells/mL (blue) and to various concentrations of noninoculated LB media in 1 M HCl solution (red) (**A**). Response corresponds to the electrocatalytic signal highlighting the bacterial detection. The curve indicates the fitting of the experimental data with a logarithmic regression: current density = 0.1377 × ln (*B. cereus* concentration) + 0.4888 (R^2^ = 0.9922) (**B**).

Moreover, a control test was performed by adding different concentrations of LB media solutions without any bacteria (noninoculated media) continuing with the same procedure (Figure 5). The incubation of this solution with the electrode resulted in a small change in the current response (less than −0.70 ± 0.05 mA/cm^2^).

The results in Figure 5 clearly indicate the detection of *B. cereus* at a concentration of 24 cells/mL. It is also noted that it is possible to detect this strain at a concentration greater than 12 cells/mL. In contrast, with a bacterial concentration of less than 12 cells/mL, no significant difference in the signal was observed compared to that obtained with the negative control represented by the sterile LB medium.

Finally, the experimental dose–response data of the *B. cereus* centri-chronoamperometric detection are adjusted with the following logarithmic regression:

Current density = 0.1377 ln (*B. cereus* concentration) + 0.4888, with a correlation coefficient R² = 0.9922.

**Figure 6:**
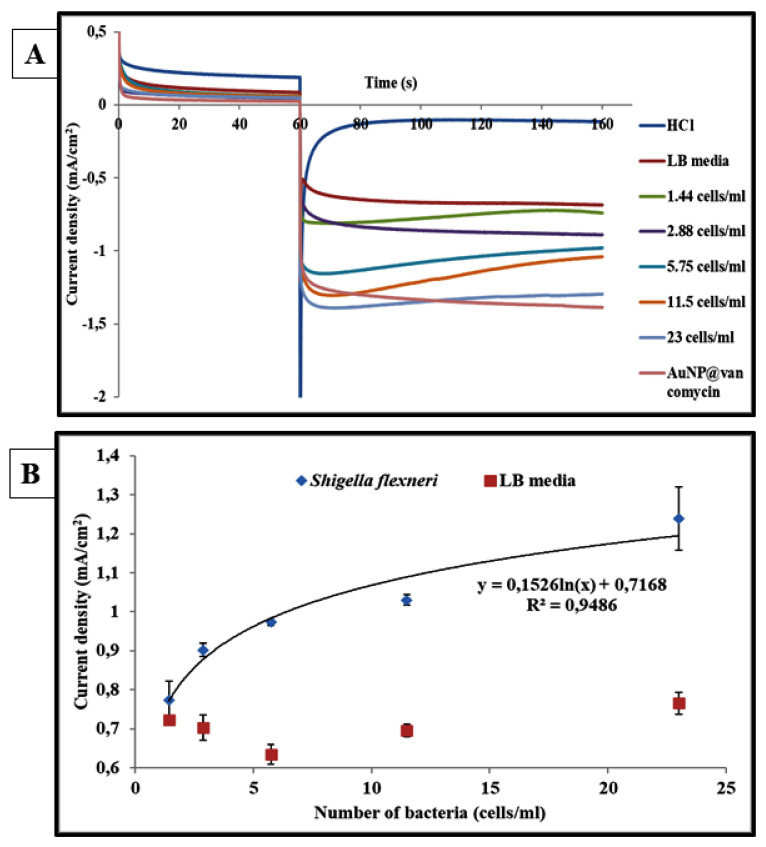
Chronoamperometric curves correspond to the response of the centri-chronoamperometric assay without bacteria (noninoculated LB media) and with various *S. flexneri* concentrations: 1.44, 2.88, 5.75, 11.5, and 23 cells/mL (**A**). Biosensor response to various concentration of *S. flexneri*. The curve indicates the fitting of the experimental data with a logarithmic regression (current density) = 0.1526 × ln (*S. flexneri* concentration) + 0.7168 (R2 = 0.9486) (**B**).

Similarly, the same method was used in order to detect the Gram-negative bacterial strain *S. flexneri*. The obtained results are presented in Figure 6. It is clearly shown that the current density increases with the concentration of this bacterium. The dose–response curve shows the possibility of detection of the *S. flexneri* strain until a concentration of 3 cells/mL. At this concentration, the generated current density was −0.90 ± 0.02 and increased until it reached 1.24 ± 0.06 at a bacterial concentration of 23 cells/mL. Therefore, the dose–response data of the *S. flexneri* detection were also adjusted with the following logarithmic regression:

Current density = 0.1526 ln (*S. flexneri* concentration) + 0.7041, with a correlation coefficient R² *=* 0.9486.

## 4. Conclusions

In conclusion, simple one-step synthesis methods of two different types of vancomycin-capped metallic nanoparticles, iron oxide and gold nanoparticles, were described. The antibiotic at the surface of these nanomaterials is able to interact with its binding sites expressed at the surface of the bacterial strain in which it has affinity. Consequently, it was clearly shown that it is possible to purify inoculated media using the superparamagnetic propriety of the coated iron oxide nanoparticles. In addition, a simple MNP@vancomycin-based agglutination assay was developed in order to confirm the existence of the bacteria and its possibility to interact with an antibiotic fixed at the surface of metallic nanoparticles. In another step, a vancomycin-functionalized gold nanoparticle-based centri-chronoamperometric assay was developed in order to rapidly detect vancomycin-sensible bacteria with high sensitivity. This method offers the possibility to evaluate the antibiotic activity on a wide range of Gram-positive and Gram-negative bacteria, but it is limited by the selectivity/specificity that it offers compared to the use of other biomolecules as bioreceptors such as monoclonal antibodies.
